# Early administration of postoperative BCAA-enriched PPN may improve lean body mass loss in gastric cancer patients undergoing gastrectomy

**DOI:** 10.1007/s00423-023-03045-6

**Published:** 2023-08-25

**Authors:** Mikiko Sakuraya, Keishi Yamashita, Michitaka Honda, Masahiro Niihara, Motohiro Chuman, Marie Washio, Kei Hosoda, Takeshi Naitoh, Yusuke Kumamoto, Naoki Hiki

**Affiliations:** 1 Department of Upper Gastrointestinal Surgery, Kitasato University School of Medicine, 1-15-1 Kitasato, Minami-Ku, Sagamihara, Kanagawa 252-0375 Japan; 2 Division of Advanced Surgical Oncology, Department of Research and Development Center for New Medical Frontiers, Kitasato University School of Medicine, 1-15-1 Kitasato, Minami-Ku, Sagamihara, Kanagawa 252-0375 Japan; 3 Department of Minimally Invasive Surgical and Medical Oncology, Fukushima Medical University, 1 Hikarigaoka, Fukushima, Fukushima 960-1295 Japan; 4 Department of Surgery, Division of Upper Gastrointestinal Surgery, Tokyo Women’s Medical University, 8-1 Kawada-Cho, Shinjuku-Ku, Tokyo, Japan; 5 Department of Lower Gastrointestinal Surgery, Kitasato University School of Medicine, 1-15-1 Kitasato, Minami-Ku, Sagamihara, Kanagawa 252-0375 Japan; 6 Department of General-Pediatric-Hepatobiliary Pancreatic Surgery, Kitasato University School of Medicine, 1-15-1 Kitasato, Minami-Ku, Sagamihara, Kanagawa 252-0375 Japan

**Keywords:** Branched-chain amino acids, Gastric cancer, Lean body mass, Nutrition therapy, Surgery

## Abstract

**Background:**

It has been reported that weight loss or lean body mass (LBM) loss after gastrectomy for gastric cancer is associated with prognosis and nutritional support alone is insufficient to prevent LBM loss. Branched-chain amino acids (BCAA) play an important role in muscle catabolism, however their clinical effects on suppression of LBM loss in gastric cancer patients undergoing gastrectomy remains elusive. In this current study, we investigated the effect of our original PPN regimen including BCAA (designated to BCAA-regimen) on LBM loss.

**Methods:**

We conducted a randomized controlled trial (RCT) at a single institution where patients undergoing gastrectomy were assigned to either receive a five-day early postoperative course of the BCAA-regimen (BCAA group) or conventional nutrition. The primary endpoint was the % reduction in LBM at postoperative day 7. The secondary endpoints included the % reduction in LBM at 1 and 3 months postsurgery.

**Results:**

At postoperative day 7, LBM loss in the BCAA group tended to be lower than in the control group (0.16% vs. 1.7%, respectively; *P* = 0.21), while at 1 month postsurgery, LBM loss in the BCAA group was significantly different to that of the control group (− 0.3% vs. 4.5%, respectively; *P* = 0.04). At 3 months postgastrectomy, however, LBM loss was similar between the BCAA and the control groups.

**Conclusion:**

Our RCT clinical trial clarified that early administration of the postoperative BCAA regimen improved LBM loss at 1 month after surgery in gastric cancer patients undergoing gastrectomy.

**Supplementary Information:**

The online version contains supplementary material available at 10.1007/s00423-023-03045-6.

## Introduction

Gastric cancer is the second leading cause of cancer deaths worldwide and is increasing as the population ages [1], putatively due to high incidence of *Helicobacter pylori* infection [2]. Probably due to this reason, the distribution of the elderly patients with gastric cancer is increasing in Japan [3]. Elderly populations are susceptible to sarcopenia, as represented by decreased muscle mass together with its functional loss. Sarcopenia is associated with short life span in the general population [4], and with poor prognosis even in patients with stage I gastric cancer after gastrectomy, reportedly linked to cancer-unrelated deaths [5, 6]. To reduce mortality of stage I gastric cancer after gastrectomy, long-term improvement of LBM may be required as in general population.

On the other hand, sarcopenia is also associated with poor prognosis in stage II/III gastric cancer who underwent standard therapy (surgery + postoperative adjuvant chemotherapy), either, putatively due to low adherence of postoperative adjuvant chemotherapy [7, 8]. Sustaining muscle mass to improve adherence to adjuvant chemotherapy for gastric cancer is therefore a critical goal for the perioperative management of patients with advanced gastric cancer who undergoing gastrectomy. This purpose might be achieved by perioperative control of LBM at 1 month after gastrectomy, because LBM loss at 1 month after gastrectomy affects continuation of chemotherapy [7]. Therefore, improvement of LBM loss 1 month after gastrectomy may improve chemotherapy adherence and long-term survival outcomes.

Nutrition is one of the most promising interventions to sustain perioperative LBM. Nutritional elements, including BCAA, have long been candidates for improving muscle metabolism in surgical patients [9, 10]. Recently BCAA administration has been actually demonstrated to improve LBM in hepatic cirrhosis with sarcopenia [11] and increase handgrip strength in the elderly general population [12]. However, such clinical effects on LBM in short-term care after gastrectomy remain unclear [13].

For effective utility of BCAA to achieve maintenance and/or improvement of LBM, sufficient energy intake with amino acid (nitrogen) should be administered [14]. According to the American Society for Parenteral and Enteral Nutrition guidelines, a non-protein calorie/nitrogen ratio (NPC/N, kcal/g) of 125 to 225 is recommended under non-stressed conditions and 70 to 100 for critically ill patients [15]. In the postoperative peripheral parenteral nutrition (PPN), however, maximal infusion is limited to 600 kcal of NPC in the 7.5% glucose infusion (for example, BFLUID including total *N* of 9.4 g/2L) to avoid phlebitis, and this energy is still not sufficient to attain the best utility of nitrogen (9.4 × 70 to 100 = 658 to 900 kcal even for critically ill patients).

In this current study, we therfore proposed that fat preparations including 500 kcal may be a promising way to achieve such sufficient energy to utilize BCAA efficiently to sustain LBM, as fat is more energy efficient (9 kcal/g) than glucose (4 kcal/g), and in addition, the concurrent administration of fat preparations is expected to reduce intravascular osmotic pressure, thereby reducing phlebitis. So, our original formula for PPN (amino acid + 7.5% glucose infusion + fat preparation including NPC of at least 1,100 kcal) is a promising regimen to improve short-term effects of LBM after gastrectomy.

Moreover, exercise is also helpful for sustaining LBM, and recent rigorous research has proposed that nutrition in combination with proper exercise can only increase LBM, whereas neither nutrition nor exercise worked on its alone [16, 17]. This finding suggests that the synergy of nutrition and exercise is critical for muscle preservation. Therefore, in this study, we conducted a clinical trial to demonstrate whether our original postoperative PPN preparations with high concentration of BCAA and fat supplementation, along with exercise rehabilitation, are effective in sustaining muscle mass during the postoperative care of patients after gastrectomy.

## Patients and methods

### Patients

This pilot study was designed as a single-center, open-labeled, superiority, randomized controlled trial (RCT) as a preliminary evaluation of the effect of BCAA administration in patients with gastric cancer who underwent gastrectomy with exercise rehabilitation. This clinical trial was registered with the University Hospital Medical Information Network Clinical Trial registry (UMIN000042579). The patients were recruited between January 2021 and October 2021 and 24 patients were prospectively enrolled in the study (C20-274).

### Randomization

Patients were randomized using a complete randomization method by a random number table. Allocation regulators were gender (male/female) and surgical procedures, consisting of two categories—distal gastrectomy/pylorus preserving gastrectomy and total gastrectomy/proximal gastrectomy.

### Sample size estimation

Sample size was calculated with reference to individual data from our previous study [18], where the postoperative LBM loss on postoperative day 7 (POD7) was − 0.2 kg in patients who immediately received infusions including amino acids (*n* = 27) and 2.2 kg in patients who were administered infusions without amino acids after gastrectomy (*n* = 49). Using this as the estimated value, with a power of 80% and a significance level of 0.05 for a one-tailed test, the minimum sample size was calculated to be 11 patients for each group. Allowing for a dropout rate of approximately 10%, we decided on a sample size of 24 patients.

### Study endpoints

The primary endpoint was the % reduction in LBM at POD7. This primary endpoint was selected, because the previous clinical trial comparing oral immunonutrition in total gastrectomy for gastric cancer had remarkable difference of LBM at POD7 between institutes that used BCAA-contaning regimens and those did not use them [18]. The secondary endpoints were as follows: % reduction in LBM at 1 and 3 months postsurgery; % reduction in both body weight (BW) and body fat mass (BFM) at POD7, 1 month, and 3 months postsurgery; physical capacity, including handgrip strength (kilogram, kg), gait speed (meter/second, m/s) assessed by a 10 m walk, and a five-repetition sit-to-stand (SS-5) test (second, s); postoperative complications (Clavien-Dindo [CD]-II or higher); postoperative inflammation status, including white blood cell count (/µ litter, µL), C-reactive protein (mg/dL), albumin (g/dL), and pre-albumin (mg/dL); and exercise adherence.

### Measurement of body composition

Measurements of body composition, such as LBM and BFM, were obtained using a InBody S10 body composition analyzer (InBody Co., Ltd., Seoul, Korea) in the sitting position. Thirty bioimpedance measurements were based on six different frequencies (1 kH, 5 kHz, 50 kHz, 250 kHz, 500 kHz, and 1000 kHz) at each body segment (right arm, left arm, trunk, rightleg, and left leg).

### Eligibility criteria

The inclusion criteria were as follows: (i) age ranging between 20 and 80 years; (ii) histologically proven adenocarcinoma of the stomach; (iii) clinical stages I-IVa (based on the 15^th^ edition of the Japanese Gastric Cancer Classification) with no distant metastasis with R0 resection; (iv) Eastern Cooperative Oncology Group performance status of 0–2; and (v) written informed consent provided. The exclusion criteria were as follows: (i) other synchronous or metachronous cancers, as well as synchronous multiple cancers in the stomach; (ii) preoperative treatment with drugs for gastric cancer; (iii) emergent surgery; (iv) cardiac disorders with a New York Heart Association functional classification of 2 or higher; (v) pulmonary disorders with a Hugh-Jones classification of 4 or higher; (vi) sufficient organ function consisting of aspartate transaminase < 100 IU/L, alanine aminotransferase < 100 IU/L, total bilirubin < 2.0 mg/dL, and serum creatinine < 1.5 mg/dL; (vii) locomotion disorders; (viii) thrombotic disorders; (ix) allergic reaction to egg, soy beans, or thiamine; (x) insulin users; (xi) mental disorders which may have affected the ability or willingness to provide informed consent or abide by the study protocol; and (xii) BCAA supplement users. After confirming that the patients met the inclusion/exclusion criteria, informed consent was obtained.

### Treatment methods

#### Nutritional support in control and intervention groups

**Fig. 1:**
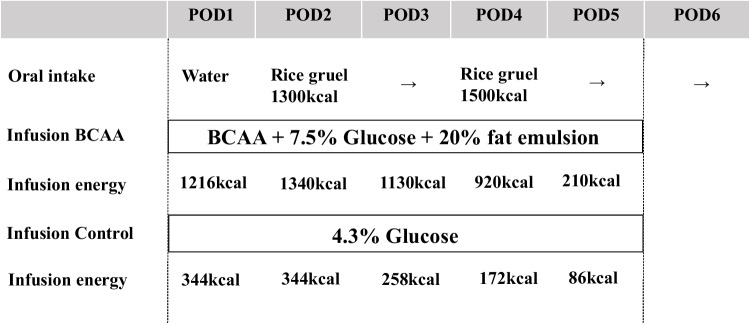
Nutrition intervention

Postoperative nutritional managements of the control group and BCAA group are shown in Fig. 1. The intervention group (BCAA group) was administered a 7.5% glucose containing BCAA (BFLUID®, Otsuka Pharmaceutical Factory, Japan) in combination with a fat preparation (500 kcal/250 mL, 20% Intralipos®, Otsuka Pharmaceutical Factory) from POD1 to POD5. The control group was administered 4.3% glucose (SOLDEM 3A®, TERUMO Corporation, Japan) from POD1 to POD5. We described details of the content in the “Results” section. Both groups were given no additional perioperative nutritional supplementation.

#### Exercise support

Rehabilitation was started from POD1 in both the control and intervention groups. The patients used a pedometer (HJ-325, Omron Corporation, Japan) to record the numbers of steps taken (100 steps at POD1, 500 steps at POD2, 1000 steps at POD3, 2000 steps at POD4, 3000 steps at POD5, 4000 steps at POD6, and 5000 steps at POD7 as their daily goals). At the time of discharge, the physiotherapist demonstrated trunk exercises that could be done at home and recommended the exercises be performed for 3 months.

#### Adjuvant treatment

Patients diagnosed with pathological stage II or III disease were given postoperative adjuvant chemotherapy using S-1. Such postoperative adjuvant therapy was initiated within eight weeks after surgery and administered for one year according to gastric cancer treatment guidelines^19^.

### Statistical analyses

Statistical analyses were performed using the SAS software package (JMP16, SAS Institute, Cary, NC). Values were initially tested for Gaussian distribution using the Shapiro–Wilk test. For Gaussian distributions, paired or unpaired two-tailed *t* tests and one-way analysis of variance were performed, followed by Tukey tests for multiple comparisons. For non-Gaussian distributions, Mann–Whitney tests and Kruskal–Wallis tests were performed, followed by Dunn tests for multiple comparisons. Unless stated otherwise, mean values and standard errors are shown. Where indicated, individual *P* values are shown.

## Results

**Fig. 2:**
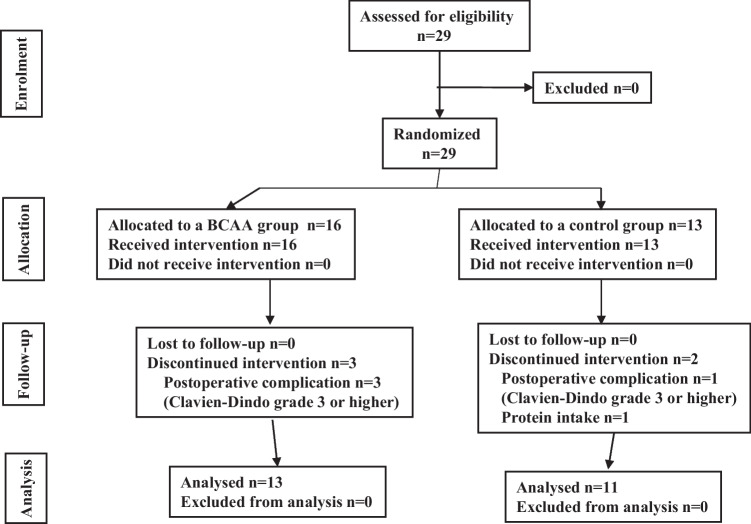
CONSORT diagram for the trial

A total of 29 patients were enrolled (Fig. 2). Of the 29 patients, 16 were randomized to a BCAA group and 13 to a control group. Postoperative nutritional managements of the control group and BCAA group are shown in Fig. 1. The intervention group (BCAA group) was administered a 7.5% glucose containing BCAA (BFLUID®, Otsuka Pharmaceutical Factory, Japan) in combination with a fat preparation (500 kcal/250 mL, 20% Intralipos®, Otsuka Pharmaceutical Factory) from POD1 to POD5. The control group was administered 4.3% glucose (SOLDEM 3A®, TERUMO Corporation, Japan) from POD1 to POD5. The infusion energies of the BCAA group were stepwise reduced as follows: POD1, 1216 kcal/day; POD2, 1340 kcal/day; POD3, 1130 kcal/day; POD4, 920 kcal/day; and POD5, 210 kcal/day. Energy administration was less at POD1 than at POD2, because BFLUID® was not used during the initial 6 hours just after surgery. The infusion energies of the control group were stepwise reduced as follows: POD1, 344 kcal/day; POD2, 344 kcal/day; POD3, 258 kcal/day; POD4, 172 kcal/day; and POD5, 86 kcal/day. The energies from food intake were the same both groups(water intake alone on POD1, maximum 1300 kcal food intake on POD2 and POD3, and maximum 1500 kcal on POD4 and POD5), in which energies from food intake on POD7 were the same as the control group. Clinicopathological and nutritional backgrounds are shown in Table 1. There was no significant difference in clinicopathological and nutritional backgrounds between the two groups.

**Table 1:** Clinicopathological and nutritional characteristics of 24 patients

Variables	BCAA group (*n* = 13)	Control group (*n* = 11)	*P* value^†^
Sex			0.2
Male	9 (69)	10 (91)	
Female	4 (31)	1 (9)	
Age, median (range), y	71 (48–78)	68 (32–79)	0.4
Body mass index, median (range), kg/m^2^	24 (18–26)	24 (21–27)	0.9
American Society of Anesthesiologists physical status (I/II/III)	0/13 (100)/0	0/9 (82)/2 (18)	0.07
Procedure			0.52
Total gastrectomy	1 (8)	2 (18)	
Distal gastrectomy	10 (77)	9 (82)	
Proximal gastrectomy	1 (8)	0	
Pylorus-preserving gastrectomy	1 (8)	0	
Approach			0.35
Laparoscopic	12 (92)	11 (100)	
Robotic	1 (8)	0	
Postoperative complication			0.44
Clavien-Dindo grade 2 or higher	1 (8)	2 (18)	
Phlebitis	0	0	
Oral intake energies (kcal/ day) at POD7	1190 (500–1500)	1200 (800–1500)	0.58
pT stage			0.52
T1a/T1b/T2/T3/T4a	5 (38)/4 (30)/2 (15)/1 (8)/1 (8)	5 (45)/3 (27)/0/3 (27)/0	
pN stage			0.36
N0/N1/N2	12 (92)/1 (8)/0	10 (91)/1 (9)/0	
pStage (JGCA)			0.57
IA/IB/IIA/IIB/IIIA	9 (69)/1 (8)/2 (15)/1 (8)/0	8 (73)/0/2 (18)/0/1 (9)	
Adjuvant chemotherapy			0.44
+	1 (8)	2 (18)	
-	12 (92)	9 (82)	

Values in parentheses are percentages unless indicated otherwise

*BCAA* branched-chain amino acid, *JGCA* Japanese Classification of Gastric Carcinoma (15th edition) [19], *POD7* postoperative day 7

^†^*χ*^2^ test

Three patients in the BCAA group with postoperative complications (CD-III or higher) were discontinued intervention and excluded from the safety analysis. Two patients in the control group with postoperative complications (CD-III or higher) and protein intake were discontinued intervention and excluded from the safety analysis.

### Reduction in LBM at POD7, one month, and three months after gastrectomy

**Fig. 3:**
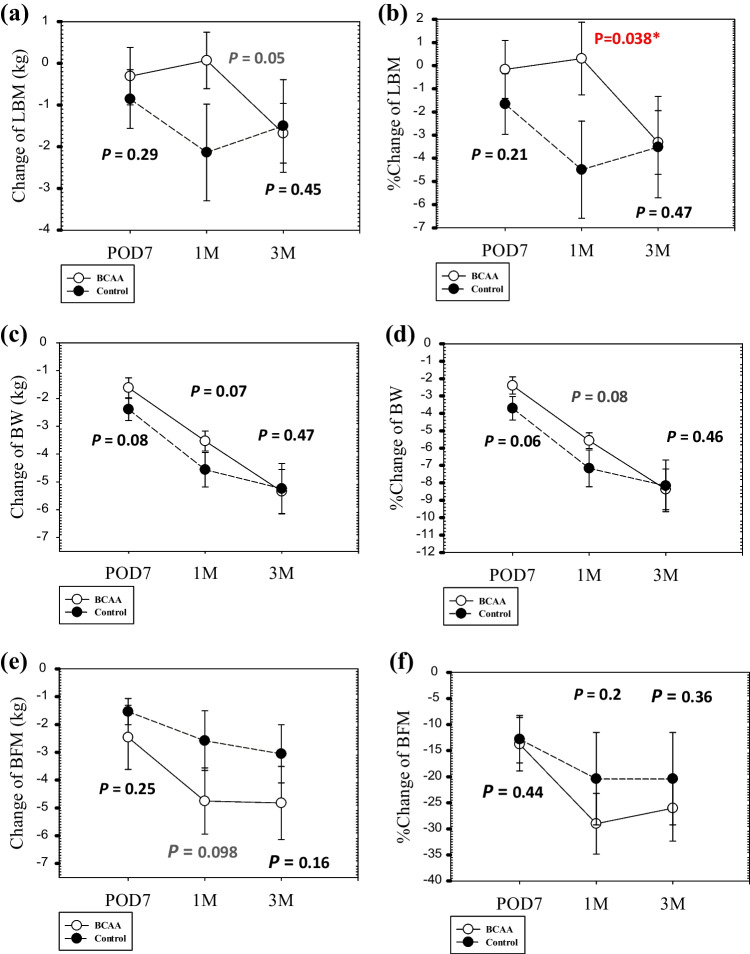
Change and % change in lean body mass (LBM), body weight (BW) and body fat mass (BFM) after gastrectomy in branched-chain amino acid (BCAA) group and control group. **a** Change of LBM. **b** %Change of LBM. **c** Change of BW. **d** %Change of BW. **e** Change of BFM; **f** %Change of BFM. ^*^P < 0.05; *t* test

The primary endpoint was a difference in the % reduction in LBM at POD7 between the BCAA intervention group and the control group. Mean LBM reduction was 0.3 kg in the BCAA group, whereas it was 0.85 kg in the control group (*P* = 0.29; Fig. 3a). After adjustment for preoperative LBM, the % reduction in LBM in the BCAA group tended to be lower than in the control group (0.16% vs. 1.7%, respectively; Fig. 3b), but there was no significant difference (*P* = 0.21). Therefore, the primary endpoint was not met.

We then investigated the secondary endpoint of a difference in the % reduction in LBM between the BCAA and control groups at 1 month and 3 months after gastrectomy. At 1 month postoperative, the mean LBM reduction was − 0.07 kg in the BCAA group, but 2.1 kg in the control group (*P* = 0.051; Fig. 3a). After adjustment for preoperative LBM, the % reduction in LBM in the BCAA group was significantly lower than in the control group (-0.3% vs. 4.5%, respectively; *P* = 0.038; Fig. 3b). At 3 months postsurgery, however, the mean LBM reduction was 1.6 kg in the BCAA group, and 1.5 kg in the control group (*P* = 0.45; Fig. 3a). After adjustment for preoperative LBM, the % reduction in LBM in the BCAA group was not significantly different to the control group (3.3% vs. 3.5%, respectively; *P* = 0.47: Fig. 3b) (Table 2).

**Table 2:** Change in LBM, BW, and BFM after operation

Variables	BCAA group (*n* = 13)				Control group (*n* = 11)			
	Preoperation	POD7	1 month	3 months	Preoperation	POD7	1 month	3 months
LBM (kg)	47.7 ± 10.3	47.4 ± 9.3	47.8 ± 10.4	46.1 ± 9.9	52.6 ± 6.9	51.7 ± 7.3	50.4 ± 8.9	51.1 ± 9.6
BW (kg)	62.9 ± 10.6	61.3 ± 9.8	59.4 ± 10.0	57.6 ± 9.8	66.0 ± 8.8	63.6 ± 9.1	61.5 ± 9.7	60.8 ± 9.4
BFM (kg)	16.3 ± 4.2	13.9 ± 4.1	11.6 ± 4.2	11.5 ± 2.9	13.5 ± 5.5	11.9 ± 5.4	10.9 ± 5.5	10.4 ± 5.0

Values are mean ± standard deviation

*BCAA* branched-chain amino acids, *POD7* postoperative day 7, *LBM* lean body mass, *BW* body weight, *BFM* body fat mass

### Reduction in BW at POD7, one month, and three months after gastrectomy

The % reduction in BW at POD7, 1 month, and 3 months after surgery was secondary endpoints in this study. At POD7, the mean BW reduction was 1.6 kg in the BCAA group, whereas it was 2.4 kg in the control group (*P* = 0.08; Fig. 3c). After adjustment for preoperative BW, the % reduction in BW in the BCAA group was slightly lower than in the control group (2.4% vs. 3.7%, respectively; *P* = 0.08; Fig. 3d).

At 1 month after the operation, the mean BW reduction was 3.5 kg in the BCAA group, and 4.6 kg in the control group (*P* = 0.07; Fig. 3c). After adjustment for preoperative BW, the % reduction in BW in the BCAA group was slightly lower than in the control group (5.6% vs. 7.2%, respectively; *P* = 0.08; Fig. 3d).

At 3 months after the operation, the mean BW reduction had increased to 5.3 kg in the BCAA group, similar to 5.2 kg in the control group (*P* = 0.47; Fig. 3c), and the % reduction in BW was no different between BCAA and control groups (8.4% vs. 8.2%, respectively; *P* = 0.46; Fig. 3d).

### Reduction in BFM at POD7, one month, and three months after gastrectomy

The % reduction in BFM at POD7, one month, and three months after surgery was also included as secondary endpoints in this study. At POD7, the mean BFM reduction was 2.5 kg in the BCAA group, and 1.5 kg in the control group (*P* = 0.25; Fig. 3e), and after adjustment for preoperative BFM, the % reduction in BFM was no different between BCAA and control groups (13.7% vs. 12.8%, respectively; *P* = 0.44; Fig. 3f).

At 1 month after gastrectomy, the mean BFM reduction was 4.8 kg in the BCAA group, and 2.6 kg in the control group (*P* = 0.098; Fig. 3e), and the % reduction in BFM was no different between BCAA and control group (29.8% vs. 20.4%, respectively; *P* = 0.2; Fig. 3f).

At 3 months after gastrectomy, the mean BFM reduction was 4.8 kg in the BCAA group, similar to 3.1 kg in the control group (*P* = 0.16; Fig. 3e). The % reduction in BFM was also no different between the BCAA and controls groups (26% vs. 22%, respectively; *P* = 0.36; Fig. 3f).

### Changes in handgrip strength, gait speed, and SS-5 test at POD7, one month, and three months after gastrectomy

Changes in handgrip strength, gait speed, and SS-5 test results at POD7, 1 month, and 3 months postgastrectomy were also included as secondary endpoints in this study. At POD7, 1 month, and 3 months postsurgery, there was no difference in the mean change in handgrip strength between the BCAA and control groups (POD7, − 0.53 vs. − 2.46 kg, *P* = 0.08; 1 month, + 1.27 vs. + 0.22 kg, *P* = 0.22; 3 months, + 0.06 vs. − 0.25 kg, respectively, *P* = 0.44; Fig. S1a).

Likewise, there was no change in gait speed or SS-5 test results at POD7, 1 month or 3 months postoperation (gait speed: POD7, + 0.05 vs. − 0.06 m/s, *P* = 0.09; 1 month, + 0.25 vs. + 0.16 m/s, *P* = 0.14; 3 months, + 0.30 vs. + 0.19 m/s, respectively, *P* = 0.14; Fig. S1b; SS-5 test: POD7, − 0.61 vs. + 0.31 s, *P* = 0.1; 1 month, − 0.98 vs. + 0.41 s, *P* = 0.06; 3 months, − 0.65 vs. − 0.72 s, respectively, *P* = 0.47; Fig. S1c).

### Complications after gastrectomy

As shown in Table 1, complications (CD classification of 2 or higher) were found in the BCAA group (*n* = 1) and in the control group (*n* = 2), and the frequencies were not different between the two groups. In the BCAA group, one patient developed an intraabdominal abscess (CD IIIB), and underwent re-operation by laparoscopy to place a drainage tube. In the control group, the complications were food stasis (CD II, *n* = 1) and pancreatic fistula (CD II, *n* = 1), with the former improved by PPN treatment, and the latter recovered with PPN plus antibiotic treatment.

### Postoperative nutrition status and inflammation status at POD7, one month, and three months after gastrectomy

As shown in Supplementary Table 1, at POD7 after gastrectomy, mean (standard deviation [SD]) serum levels of albumin were 3.3 (0.4) and 3.5 (0.5) g/dL in the BCAA and control groups, respectively, which were no different (*P* = 0.159). At 1 month after gastrectomy, mean (SD) serum levels of albumin were 3.9 (0.3) g/dL in the BCAA group, no different to 4.1 (0.5) g/dL in the control group (*P* = 0.353). At 3 months after gastrectomy, mean (SD) serum levels of albumin were also no different between groups (4.0 [0.2] vs. 4.1 [0.5] g/dL, BCAA vs. control, respectively *P* = 0.259).

At 7 POD, 1 month, and 3 months after gastrectomy, there was no difference in the mean (SD) serum levels of prealbumin between the BCAA group and the control group (POD7, 16.9 [4.0] vs. 16.4 [3.9] mg/dL, *P* = 0.372; 1 month, 20.7 [4.1] vs. 20.9 [2.6] mg/dL, *P* = 0.44; 3 months, 20.8 [3.9] vs. 22.5 [5.2] mg/dL, respectively, *P* = 0.202).

Finally, there were no significant differences in the mean number of white blood cells at POD7, 1 month, and 3 months postgastrectomy (*P* = 0.209, *P* = 0.059, and *P* = 0.195, respectively), or in the mean serum levels of C-reactive protein at POD7, 1 month, and 3 months after surgery (*P* = 0.178, *P* = 0.302 and *P* = 0.412, respectively).

### Compliance with exercise support

The completion rate for the exercise program was 100%; that is, all enrolled patients recorded both the number of steps taken using the pedometer and the trunk exercises performed in the action diary for 3 months after gastrectomy. The mean (SD) accumulated number of steps up to POD7 was 3314 (1607) steps in the BCAA group, similar to 3757 (1595) steps in the control group (*P* = 0.26). The mean (SD) days of walking exercise up to 3 months after gastrectomy was 85.5 (4.2) days in the BCAA group and 87.1 (4.0) days in the control group, which was not different between groups (*P* = 0.19).

## Discussion

Previous reports theoretically proposed that postoperative administration of BCAA may be effective in preventing muscle catabolism in terms of nitrogen balance [9]; however, our RCT is the first report that BCAA-regimen can actually suppress LBM loss during the postoperative course after gastrectomy. Surprisingly, in this current trial, only a five-day treatment with our original PPN, including BCAAs, in the early postoperative period significantly improved LBM loss at 1 month after gastrectomy. At POD7, the average increase in LBM by BCAA administration was 0.5 kg, while at 1 month it was 2.2 kg. On the other hand, this difference was almost eliminated to only 0.1 kg at 3 months after the operation.

Sufficient energies have been considered to optimize the effects of BCAA on nitrogen (N) retention that may represent suppression of LBM loss [20–22], and NPC/N ratio of 163 was actually shown to be better than an NPC/N ratio of 102 in terms of BW reduction in stable patients without sepsis [20]. In order to minimally suppress LBM loss, we used a combination of commercial-based nutrition formulae (BFLUID® + Intralipos®) that has 1,100 kcal/day of NPC with an NPC/N ratio of 117 including 9.5 g/day of BCAA for our postoperative PPN. Clinical situation of the patients after gastrectomy are consideded between stable conditions and critically ill patients, and sufficient energies may be required beyond 1000 kcal in this study in constrast to control preparations (340 kcal/day).

The BCAA (leucine, isoleucine, and valine) are components of skeletal muscle, and they are involved in the promotion of protein synthesis and suppression of its degradation, representing protein anabolism [22]. BCAA are degraded by branched-chain aminotransferase (BCAT) mainly in the intracellular mitochondria and converted to branched-chain alpha-ketoacids. Such metabolites from leucine, for example, include beta-hydroxy-beta-methylbutyric acid (HMB) that outstandingly promotes protein synthesis [22].

BCATm (mitochondria type), which encodes the enzyme catalyzing the first step in peripheral BCAA catabolism and BCAA, in blood is high in BCATm knockout mice, in which BFM and BW are decreased even though increased food intake [23]. Among the BCAA, leucine (Leu) has been repoted to directly stimulate mTOR signling in the hypothalamus, leading to decrease food intake [24]. In addition, Leu may influence satiety by stimulating leptin secretion [25]. Moreover, dietary supplements of Leu or BCAA have been shown to decrease BFM and BW [26] and to improve glucose metabolism in some cases [27]. These findings suggest that BCAA supplements may be beneficial in control of BFM.

In the present study, we therefore interpreted the clinical efficacy of BCAAs as actually being confirmed in patients after gastrectomy, since not only LBM was maintained but also BFM was reduced in the BCAA group. We do not consider the reduction in BFM to be an undesirable clinical response, as it is thought to have a favorable effect on decreasing insulin resistance and controlling blood glucose levels.

In our study, we demonstrated that our original early postoperative nutritional enhancement regimen may be effective in maintaining muscle mass after gastrectomy. The clinical significance of maintaining of muscle mass after gastrectomy differs in early and advanced gastric cancer. The former is expected to be related to a possible reduction of deaths from other diseases, while the latter is anticipated to provide oncological benefits of improving adherence to adjuvant chemotherapy.

Our treatment regimen was able to achieve increasing of LBM at 1 month after gastrectomy, but was not able to maintain this effect until 3 months after gastrectomy. Hence, early postoperative nutritional enhancement alone may not be sufficient for such long-lasting maintenance of LBM staus beyond 3 months after surgery. On the other hand, the possibility of maintaining LBM after 1 month after gastrectomy by nutritional enhancement only in the early postoperative period was herein proposed, hence which could serve as an adjuvant chemotherapy to enhance the effect of adjuvant chemotherapy in advanced gastric cancer. In fact, it has been reported that the LBM loss after gastrectomy is associated with continuation of adjuvant chemotherapy and even affects prognosis [28].

In our current clinical trial, all the patients were required to take part in the postoperative exercise rehabilitation program. In maintaining musculoskeletal health, exercise has been shown to increase the sensitivity of even senescent muscle to the anabolic properties of protein-based nutrition [29]. In community-dwelling elderly sarcopenic women, both nutrition therapy and exercise therapy improves sarcopenia, whereas either therapy alone does not [16]. Taking these facts into account, we must note that our clinical success is conditional on patient exercise compliance.

Our current study still has limitations. First, the patient number was small and the study was conducted at a single institute, however the patients’ number was determined based on the pertinent statistical processes instructed by professional medical statistician (MH). Second, while the patients of this study had few complications, such complications may have affected the final outcomes. Third, our original BCAA-regimen group was different from the control group in terms of fat emulsion and glucose concerntration in addition to BCAA, and the final effect might be obtained by factors other than BCAA.

In conclusion, after gastric cancer surgery, a five-day early postoperative intervention of BCAA administration does not significantly suppress the % reduction in LBM at 7 days, but it has a great potential to suppresse the % reduction in LBM at 1 month. Based on these promising study outcomes, we will conduct a multi-institutional larger trial to validate % reduction in LBM at 1 month after gastrectomy for gastric cancer in the near future.

### Supplementary Information

Below is the link to the electronic supplementary material.

Supplementary file1 (ZIP 65 KB)

## Data Availability

The authors confirm that the data supporting the findings of this study are available within the article and/or its supplementary materials.

## Declarations

### Animal studies

N/A.
