# Supplementary material for: Early administration of postoperative BCAA-enriched PPN may improve lean body mass loss in gastric cancer patients undergoing gastrectomy
Source: Langenbecks Arch Surg. 2023 Aug 25;408(1):336. doi: 10.1007/s00423-023-03045-6 (PMC10457225; doi:10.1007/s00423-023-03045-6)
Supplement: Supplementary file 1 — Supplementary file1 (ZIP 65 KB) [file 423_2023_3045_MOESM1_ESM.zip › 22j5372R_Fig.S1 (4).pptx]

## Slide 1
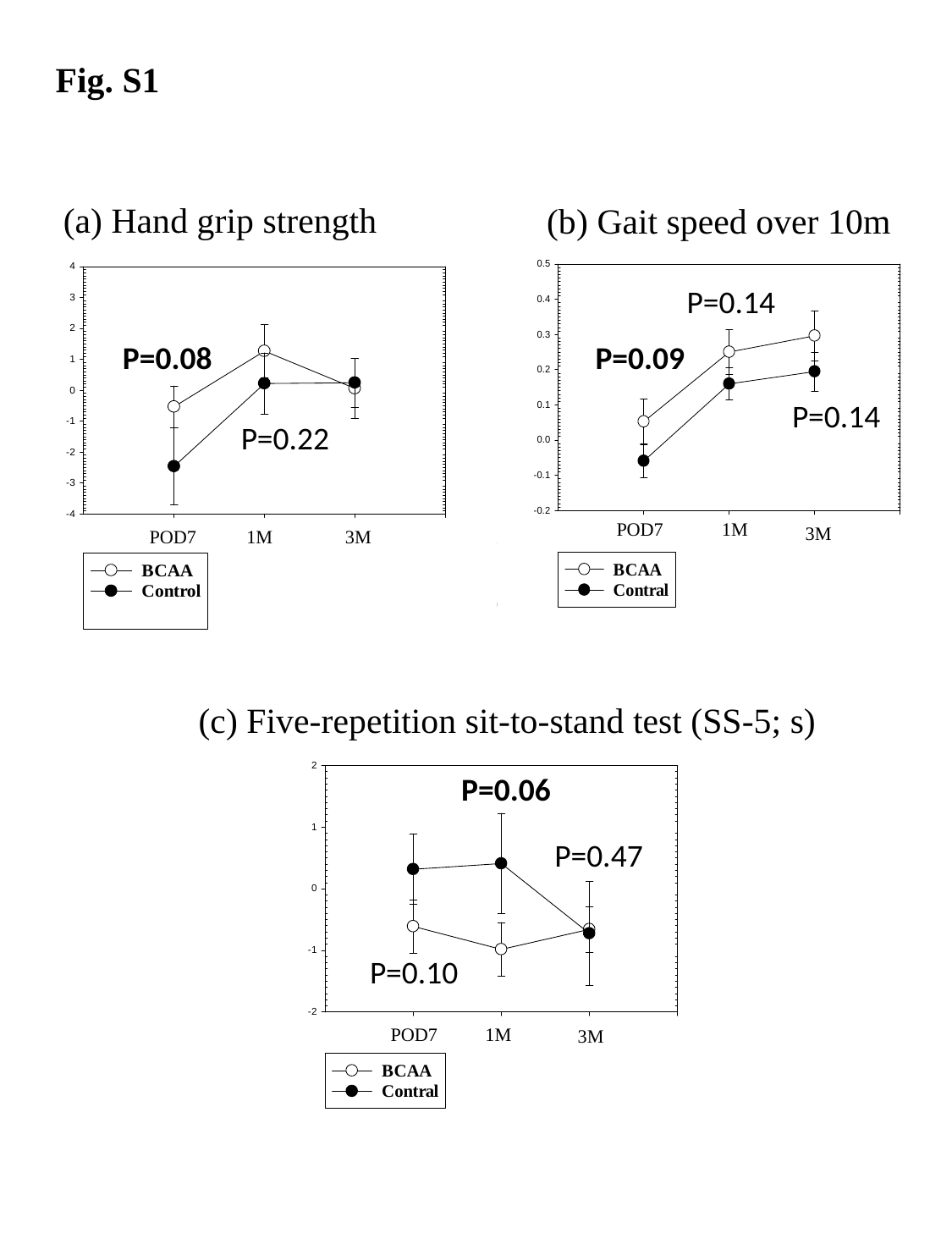

Fig. S1
(a) Hand grip strength
P=0.08
P=0.22
3M
POD7
1M
(b) Gait speed over 10m
P=0.14
P=0.09
P=0.14
1M
POD7
3M
P=0.44
(c) Five-repetition sit-to-stand test (SS-5; s)
P=0.06
P=0.47
P=0.10
1M
POD7
3M
